# Comparison of Binaural Tone Music vs Patient Choice Music vs Midazolam on Perioperative Anxiety in Patients Posted for Surgery Under Spinal Anaesthesia: a Randomized Control Trial

**DOI:** 10.7759/cureus.35091

**Published:** 2023-02-17

**Authors:** Markandey Prasad, Priyanka Sethi, Kamlesh Kumari, Ankur Sharma, Manbir Kaur, Pawan K Dixit, Pradeep Bhatia, Deepanshu Dang, Shipra Roy, Nisha MP

**Affiliations:** 1 Anaesthesiology and Critical Care, All India Institute of Medical Sciences, Jodhpur, Jodhpur, IND; 2 Burns and Plastic Surgery, All India Institute of Medical Sciences, Jodhpur, Jodhpur, IND

**Keywords:** perioperative anxiety, pain scores, spinal anaesthesia, perioperative music, binaural tone music

## Abstract

Background

Perioperative anxiety affects patients' hemodynamics by increasing stress levels, leading to delayed recovery. In this study, we compared the anxiety-reducing effect of music (patient choice and binaural tone music) with midazolam for perioperative anxiolysis in patients undergoing surgery under spinal anaesthesia.

Methods

After obtaining institutional ethical clearance and informed written consent, a total of 225 patients classified as ASA grades 1 and 2 (American Society of Anesthesiologists) were enrolled and randomised into three groups of 75 patients per group. Group A patients received research-selected music (binaural tone) via noise-cancelling headphones, Group B received intravenous midazolam (minimum of 1 mg to 2 mg maximum) as per clinical judgement, and Group C participants provided patient-preferred music via noise-cancelling headphones. The patient's perioperative anxiety was assessed using a visual analogue anxiety scale at regular time intervals.

Results

Anxiety scores were significantly reduced in the patient's choice music group (Group C) and binaural tone music group (Group A) as compared to the midazolam group (Group B). Postoperative pain scores were statistically significantly lower in Group C, followed by Group A and Group B. On comparing patient satisfaction scores, using numerical rating scores, 96% of patients in Group C achieved excellent scores with a p-value of 0.007.

Conclusion

Binaural tone music and patient choice music can be suitable alternatives to pharmacological therapies for perioperative anxiolysis.

## Introduction

Preoperative anxiety can significantly affect a patient’s perioperative course by elevating stress markers, promoting fluctuations in hemodynamics, and thus negatively impacting postoperative recovery [1,2]. It can be associated with abnormal hemodynamic changes because of sympathetic, parasympathetic, and endocrine stimulation.

Preoperative anxiety is routinely treated with short-acting benzodiazepines, which have few associated side effects. Nowadays, the focus is shifting from pharmacological to non-pharmacological interventions [3]. Music therapy is a non-pharmacological intervention, virtually harm-free and cost-effective. Music can be of different types of pitch, rhythm, or tone colour. The literature has shown that high-pitched music elicits stress, while low-pitched, slow-paced, orchestral, harmonious music and music beats similar to a human heartbeat (60-80 beats/min) provide relaxation and have sound therapeutic effects when listened to for at least 30 min [4,5].

Binaural beats are the perception of sound created by the brain when two different tones of different frequencies (<1500 Hz) are heard simultaneously in both ears. When listened to for a period of time, binaural beats can synchronize with brain waves and alter brain wave activity as well as one's levels of arousal [6]. The rhythm of the binaural beat is the difference between the two frequencies. Various binaural beat-infused music styles can simulate the alpha, theta, and delta EEG waveforms. The binaural beat frequency can thus be selected to produce EEG-associated states. The difference between the two frequencies must be small (below about 30 Hz) for the effect to occur. For example, if a 200 Hz sine wave is played into the right ear and a 210 Hz into the left ear, the brain is entrained towards the beat frequency of 10 Hz, in the alpha range, which is associated with relaxation [7,8]. We choose a freely available version of binaural tone music specifically meant for anxiolysis [9]. The aim of this study was to investigate and compare the anxiety-reducing effect of music (patient choice and binaural tone music) with midazolam, played preoperatively from 10 min before administration of spinal anaesthesia till 30 minutes postoperatively.

## Materials and methods

This prospective randomised control study was conducted in the Department of Anaesthesia and Critical Care at All India Institute of Medical Sciences (AIIMS), Jodhpur, from January 2020 to June 2021. Institutional ethics committee approval (AIIMS/IEC/2019-20/1000) and Clinical Trials Registry-India (CTRI) registration (CTRI/2020/03/023932) was obtained prior to the commencement of the study. Patients belonging to the American Society of Anaesthesiologists (ASA) physical status grade I and II between 18 to 60 years of age scheduled to undergo surgery under spinal anaesthesia were included. Patients who refused to give consent, or those who were pregnant, breastfeeding, or had a history of psychiatric illness, hearing impairments or chronic pain were excluded from the study. 

**Figure 1:**
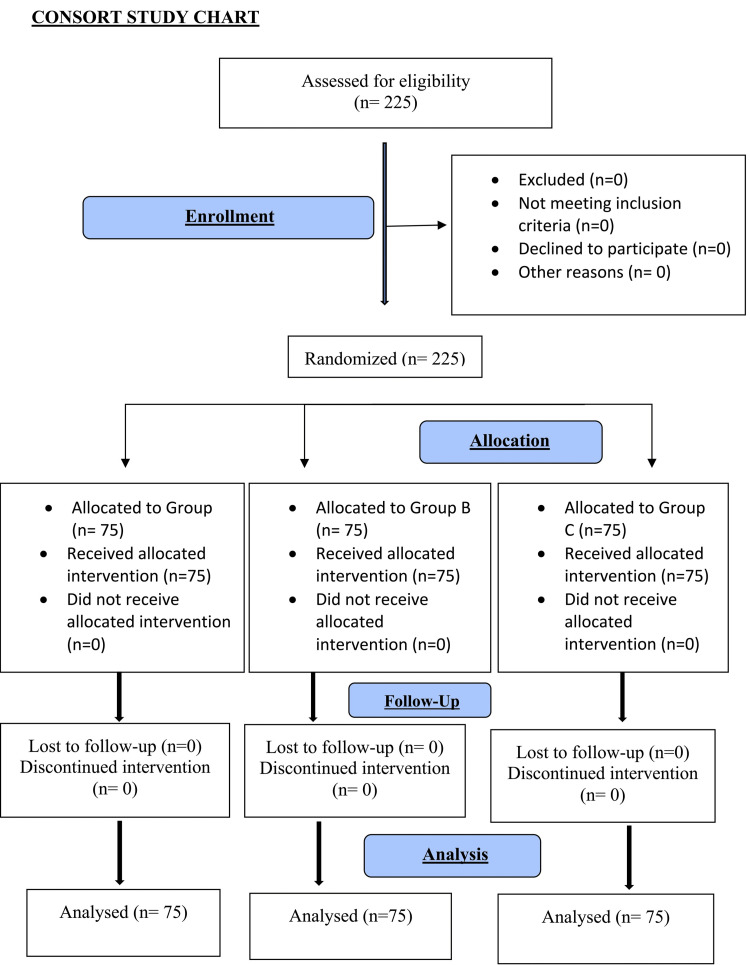
CONSORT diagram CONSORT flow chart

All the patients were examined one day before surgery by the attending anaesthesiologist during the preoperative visit. After providing written informed consent and confirming fasting status, 225 patients were randomly allocated into three groups of 75 patients per group in a one-to-one fashion using a computer-generated algorithm into three groups namely Group A, Group B and Group C (Figure 1).

A simple random sequence was generated, and the group allocation numbers were concealed in sealed opaque envelopes which were opened just before the spinal anaesthesia procedure. Group A patients received binaural tone (frequency 3.5 Hz) music via noise-cancelling headphones, Group B received intravenous midazolam (minimum of 1 mg to 2 mg maximum) as per clinical judgement, and Group C patients were made to listen to patient choice music via noise-cancelling headphones. A staff member otherwise uninvolved with the patients opened the sealed envelope containing the randomized treatment, i.e. binaural tone music (Group A), patient choice music (Group C) or control group (no sound but intravenous midazolam; Group B). The same staff member was also made to fit the in-ear earphones into the patient’s ears and adjust the volume on the smartphone controlling the sound. All patients, including the control group, wore earphones during the procedure to ensure the blinding of the staff. The patients were also blinded. They were given the freedom to adjust and readjust the volume of music during the entire listening experience. Anxiety was assessed by using visual anxiety score (VAS-A) which comprises a 10 cm line on which the participant marks their current degree of anxiety with the left end of the line being labelled as no anxiety or calm and the right end being labelled as maximum anxiety. A similar scale was used for assessing the severity of pain (0 to 10 cm) with the left side representing no pain and the extreme right representing the maximum severity of pain imaginable. In the preoperative area, the research assistant assessed the VAS-A score. In the operating room, routine monitoring processes were conducted including continuous electrocardiography (ECG), non-invasive blood pressure (NIBP), and peripheral oxygen saturation (SpO2). Baseline vitals were recorded. At this point, patients received either intravenous midazolam or started listening to music based on their group assignment. Patients in the midazolam group wore headphones without music and normal saline was given by syringe as a bolus in music groups to maintain blinding. The patients' anxiety scores were reassessed 10 minutes after intervention, before and after giving spinal anaesthesia. The patient listened to music throughout the surgery; intraoperatively, VAS-A was assessed at 60 minutes, then at skin closure. The patient continued to receive intervention throughout the surgery till 30 minutes postoperatively. Heart rate (HR), SpO2, and blood pressure were continuously monitored throughout the surgery.

In the postoperative period, VAS-A was again assessed at 30 minutes. Postoperative pain was evaluated by an independent observer using a visual analogue score (VAS) with a 0-10 scale (0=no pain and 10=worst imaginable pain), first immediately post-operation, and then at 30-minute intervals over the next two hours. The time to first rescue analgesia was also recorded. At 24 hours after surgery, the satisfaction of the patients was assessed using a numerical satisfaction score from four to one. Four and three were considered excellent and good results, respectively, while scores of two and one indicated fair and poor results.

Statistical analysis

The sample size was calculated using data from the study by Arslan et al [10]. Post-intervention mean and standard deviation (SD) VAS scores were 2.73 and 1.28 in the music group and 3.61 and 1.4 in the control group respectively. A sample size of 75 per group was calculated by taking a 95% confidence interval, 95% power, and 20% contingency. 

Mean ± SD was used to describe the continuous data. Median ± interquartile range (IQR) was used to describe ordinal or non‐normally distributed data. One-way analysis of variance (ANOVA) was used to compare the mean value of variables in all three groups. Post-hoc analysis was done using Games-Howell or Tukey’s test, depending on the normality of the data. The difference between proportions was analyzed using the chi-square test. A p-value of <0.05 was taken as significant.

## Results

Baseline characteristics were similar in all three groups (Table 1).

**Table 1:** Demographic profiles of the study population Group A: binaural tone music; Group B: midazolam; Group C: patient choice music SD: standard deviation; M: male; F: female; ASA: American Society of Anaesthesiologists; NS: non-significant

Parameters	Group A	Group B	Group C	P-value
Age (Mean ± SD)	37.68 ± 12.33	35.65 ± 12.27	34.75 ± 12.39	0.972 (NS)
Sex (M/F)	49/26	58/17	59/16	0.124 (NS)
ASA grade (I/II)	35/40	33/42	42/34	0.231 (NS)
Duration of surgery (hours) (Mean ± SD)	3 ± 0.5	2.9 ± 0.6	3.2 ± 0.7	0.832 (NS)

Baseline anxiety scores were comparable in all groups. Among all three groups, there was a statistically significant difference between the mean VAS anxiety scores after 10 minutes, before block placement, after block placement, and at 60 minutes after the block placement (Table 2).

**Table 2:** Comparison of VAS-A among the study group Group A: binaural tone music; Group B: midazolam; Group C: patient choice music * significant VAS-A: visual analogue scale-anxiety; SD: standard deviation; NS: non-significant

VAS-A score timings	Group A (Mean ± SD)	Group B (Mean ± SD)	Group C (Mean ± SD)	P-value
Baseline	4.72 ± 1.36	4.75 ± 1.13	4.44 ± 1.05	0.218 (NS)
After 10 minutes	3.35 ± 1.36	4.53 ± 1.07	3.80 ± 1.08	0.0005*
Before spinal anaesthesia	4.00 ± 1.23	4.28 ± 0.88	3.71 ± 1.00	0.004*
After spinal anaesthesia	3.31 ± 1.30	3.90 ± 0.97	3.48 ± 0.94	0.035*
At 60 minutes	3.20 ±1.14	3.00 ± 0.82	3.35 ± 0.74	0.038*
Skin closure	2.24 ± 1.22	3.04 ± 0.86	2.47 ± 0.91	<0.001*
After 30 Minutes	2.16 ± 1.26	2.76 ± 0.73	2.21 ± 0.70	<0.001*

On intragroup comparison, using one-way ANOVA, there was a significant difference in anxiety scores between Groups B and C, and Groups A and B. There was no statistically significant difference between Group A and C in anxiety scores at various time points. 

**Figure 2:**
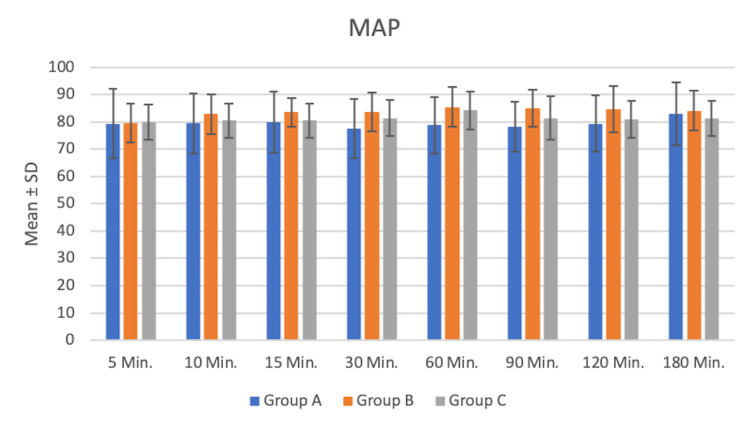
Comparison of mean arterial pressure (mmHg) among the study group Group A: binaural tone music; Group B: midazolam; Group C: patient choice music MAP: mean arterial pressure

No patient in our study group had any perioperative complications. On comparing mean blood pressures and pulse in all three groups, patients on binaural tone had statistically significant lower blood pressures and pulse rates when compared to midazolam and patient choice music groups at different time points (Figure 2).

There was no statistically significant difference in the time of rescue analgesia postoperatively in all three groups. The time of rescue analgesia (hours) in Group A was 6.96±1.61, in Group B was 7.21±1.60, and in Group C was 6.81±1.33 (P-value 0.267).

**Figure 3:**
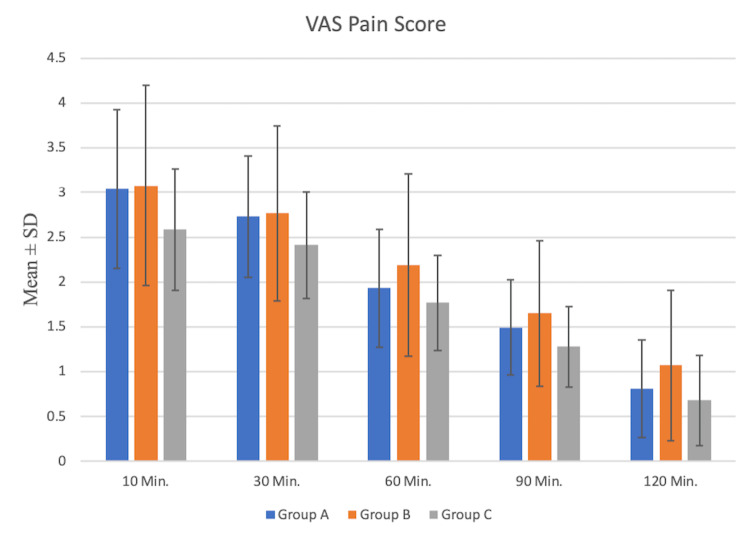
Comparison of mean VAS pain score among the study group Group A: binaural tone music; Group B: midazolam; Group C: patient choice music; m: minutes for pain assessment; VAS: visual analogue scale

Postoperative pain scores (VAS pain scores) were statistically different in all three groups (Table 3, Figure 3).

**Table 3:** Comparison of mean VAS pain score among the study group Group A: binaural tone music; Group B: midazolam; Group C: patient choice music * significant VAS: visual analogue scale; SD: standard deviation; NS: non-significant

VAS Pain score timings	Group A (Mean ± SD)	Group B (Mean ± SD)	Group C (Mean ± SD)	P value
10 minutes	3.04 ± 0.89	3.08 ± 1.12	2.59 ± 0.68	0.001*
30 minutes	2.73 ± 0.68	2.77 ± 0.98	2.41 ± 0.59	0.008*
60 minutes	1.93 ± 0.66	2.19 ± 1.02	1.77 ± 0.53	0.004*
90 minutes	1.49 ± 0.53	1.65 ± 0.81	1.28 ± 0.45	0.001*
120 minutes	0.81 ± 0.54	1.07 ± 0.84	0.68 ± 0.50	0.001*
180 minutes	0.19 ± 0.43	0.43 ± 0.68	0.04 ± 0.20	<0.001*

Post-hoc, Groups A and B had no difference, but Groups B and C had a significant difference for the next two hours. There was a statistically significant difference in Groups A and C at all time points.

Regarding the patient satisfaction score, Group A showed excellent scores in 78.67% and good scores in 21.33% of cases at 24 hours postoperatively. Group B showed excellent scores in 84% of patients and good scores in 16% of cases. Group C showed excellent scores in 96% of cases and good scores in 4% of cases with a p-value of 0.007.

## Discussion

This study was conducted to evaluate and compare the effect of different types of music, including binaural tone and patient choice music with midazolam on perioperative anxiety. Anxiety can adversely affect a patient’s perioperative course by elevating stress markers, promoting fluctuations in hemodynamics, and thus affecting postoperative recovery [11]. To our knowledge, no study has been published comparing the effect of binaural tone music with patient choice music and midazolam on perioperative anxiety as well as comparing the need for postoperative analgesia in patients posted for surgery under spinal anaesthesia. Music can modulate mood and behaviour and reduce an individual’s perception of pain through the release of endogenous opioids and interfering with nerve conduction. Binaural beats are brainstem auditory responses believed to originate in the superior olivary nucleus and have the potential to act as anxiolytic agents [6,12].

Anxiety scores were significantly lower in the group with patient choice music and binaural tone music at various time points in comparison to the midazolam group. On intragroup comparison, between binaural tone and patient choice music, anxiety scores were lower in the patient choice music group while results were identical when the patient choice music group was compared with midazolam. These results are probably due to patients being more distracted and involved with the music of their choice. 

Similarly, this study is concordant with studies by Padmanabhan et al. [7] and Roshani et al. [13], who proved that binaural tone music has significant potential to reduce preoperative anxiety and pain, thus maintaining hemodynamic parameters. On comparing intraoperative mean arterial pressure (MAP) and pulse rates between binaural tone music and the midazolam group, MAPs were significantly lower in binaural tone music at different time points. When the patient's choice of music was compared with midazolam, the music group had lower MAPs. This could be attributable to greater relaxation and anxiolysis achieved by music therapy (both binaural tone and patient choice music). This finding is comparable to the study conducted by Kahloul et al. [14] which concluded that there was more stability in mean systolic blood pressure among the music group when compared with the control group. Muddana et al. [15] found significant reductions in anxiety scores perioperatively along with postoperative blood pressures in the music group for patients undergoing cataract surgeries. Ölçücü et al. [16] also proved that listening to pure binaural beats may be a simple and effective method to reduce anxiety levels and pain.

In this study, postoperative pain scores using the VAS score were also assessed in all three groups at different time intervals. Postoperative pain scores were significantly lower in the patient-selected music group, followed by binaural tone music and the midazolam group. This study is in contrast with the study conducted by Patiyal et al. [17], who concluded that music did not affect postoperative opioid consumption. Our study concords with the study by Kühlmann et al. [18], who proved a reduced need for postoperative analgesics in patients posted for general anaesthesia. Kwon et al. [19] also found lower pain scores in patients listening to music perioperatively similar to our study. The reason for reduced pain in this study is that the patients in the preferred selected music group were more distracted and less anxious than those in other groups; this might reduce the effects of some factors, especially psychological which can lead to enhanced pain perception in the perioperative period. Moreover, music therapy may also release some endorphins, reducing pain perception [20,21]. None of the patients in this study received intraoperative and postoperative dexmedetomidine infusions.

On comparing the time of rescue analgesia after surgery, it was comparable in all three groups. It is similar to previous literature on music's effect on postoperative analgesic requirements. Patiyal et al. [17] proved that music does not make any change in the time of rescue analgesic, similar to our findings.

On comparing patient satisfaction rates in all three groups, patients listening to the music of their choice, were maximally satisfied. Almost 96% of patients had excellent satisfactory scores in this group compared to midazolam (84%) and binaural tone music (59%). This study has confirmed the beneficial effects of music therapy on patient satisfaction. Music therapy improves satisfaction directly through its relaxing effect and indirectly through its impact on other dissatisfaction factors such as perioperative pain and stress and postoperative nausea and vomiting. Surgical time was similar in all three groups and no patient in any of the groups had any significant complication.

A few limitations are also associated with this study. We used a visual analogue scale, which is a subjective scoring system, to assess anxiety and pain. We could have added more objective scales such as state-trait anxiety inventory (STAT) scoring scales [22] to generalize the results. We involved patients posted under spinal anaesthesia in our study. If patients with all types of regional anaesthesia were involved, the results would have been more generalized with more applicability. EEG recording was not done during exposure to binaural beats, which may have helped to understand how and to what extent music can affect anxiety. 

## Conclusions

Music therapy is a non-invasive, cost-effective therapy that can add a new dimension to the perioperative anxiety management of patients. Binaural tone music and patient choice music had comparable effects on perioperative anxiety. Patient satisfaction score was better in the patient choice music group. Music can be a suitable alternative to pharmacological therapy for anxiolysis in patients undergoing surgery under spinal anaesthesia. Binaural tone music had no superiority over patient choice music in terms of its role as an anti-anxiolytic. However, randomized control trials with a larger sample size are needed to establish our study's results further. 
